# Supplementary material for: Does childhood experience of family victimization influence adulthood refusal of wife abuse? Evidence from rural Bangladesh
Source: PLoS One. 2021 Jun 3;16(6):e0252600. doi: 10.1371/journal.pone.0252600 (PMC8174681; doi:10.1371/journal.pone.0252600)
Supplement: S1 Table — (PDF) [file pone.0252600.s001.pdf]

S1 Table. Items used in the outcome and main exposure variables

Outcome Variable: Items of the attitudinal refusal/acceptance of wife abuse scale

Whether a husband should abuse his wife if she does the followings:

1. Burning the food during cooking
2. Failing to prepare meals on time
3. Failing to prepare delicious dishes
4. Getting involved in an extra-marital affair
5. Arguing with the husband all the time
6. Chatting with a man disliked by her husband

Responses: 0 = reject abuse, 1 = accept – emotional abuse, 2 = accept – physical abuse, 3 = accept – both emotional and physical abuse

Scale total score: Range: 0 –to– 18

Classification: Refusal of overall wife abuse: 0 = Refused, 1 – 18 = Not refused

Classification of the refusal of wife abuse on disobeying family obligations:

**Factor 1 - Disobeying family obligations**	**Refused**	**Accept Emotional**	**Accept Physical**	**Accept Both**
• Burning the food during cooking	0	1	2	3
• Failing to prepare meals on time	0	1	2	3
• Failing to prepare delicious dishes	0	1	2	3
**Total score=**	**Range: 0 –to– 9**			
Classification of summated score=	0 = Refused, 1 – 9 = Not refused			

Classification of the refusal of wife abuse on challenging male authority:

**Factor 2 - Challenging male-authority**	**Refused**	**Accept Emotional**	**Accept Physical**	**Accept Both**
• Getting involved in an extra-marital affair	0	1	2	3
• Arguing with the husband all the time	0	1	2	3
• Chatting with a man disliked by her husband	0	1	2	3
**Total score=**	**Range: 0 –to– 9**			
Classification of summated score=	0 = Refused, 1 – 9 = Not refused			

Classification of the refusal of emotional wife abuse (recoded item responses):

**Emotional wife abuse**	**Refused**	**Accept Emotional**	**Accept Physical**	**Accept Both**
• Burning the food during cooking	0	1	0	2
• Failing to prepare meals on time	0	1	0	2
• Failing to prepare delicious dishes	0	1	0	2
• Getting involved in an extra-marital affair	0	1	0	2
• Arguing with the husband all the time	0	1	0	2
• Chatting with a man disliked by her husband	0	1	0	2
**Total score=**	**Range: 0 –to– 12**			
Classification of summated score=	0 = Refused, 1 – 12 = Not refused			

Classification of the refusal of physical wife abuse (recoded item responses):

Physical wife abuse	Refused	Accept Emotional	Accept Physical	Accept Both
• Burning the food during cooking	0	0	1	2
• Failing to prepare meals on time	0	0	1	2
• Failing to prepare delicious dishes	0	0	1	2
• Getting involved in an extra-marital affair	0	0	1	2
• Arguing with the husband all the time	0	0	1	2
• Chatting with a man disliked by her husband	0	0	1	2
**Total score=**	**Range: 0 –to– 12**			
Classification of summated score=	0 = Refused, 1 – 12 = Not refused			

Exposure Variable: Childhood experience of emotional and physical abuses

Has anyone in your family done the followings with you at childhood?

Emotional abuse

Mild abuse

1. -Insulted or swore at you, shouted or yelled at you
   -Said something to spite you, verbally rebuked at you, called you ugly

2. -Restricted you to go out of home, resisted you to contact with your friends
   -Restricted you to attend school, restricted you to participate in sports/entertainments

Severe abuse

3. -Threatened you to kill or injure you seriously
   -Did anything with you so that you got so frightened
   -Did anything with your sibling/mom so that you got so frightened

Physical abuse

Mild abuse

4. -Thrown something heavy to hurt you
   -Twisted your arm or hair, slapped you
   -Pushed, slammed, grabbed, shoved you

Severe abuse

5. -Punched you, kicked you
   -Beaten up you with stick or something else

6. -Choked you with pillow or other way
   -Burned you with something hot/fire
   -Thrown at you hot water/oil/something else
   -Hit you with knife or something sharp things

Scoring: 0 = none (non-exposed to any items)
1 = mild (exposed to mild items only)
2 = severe (exposed to severe items or severe+mild items)
