# Supplementary material for: Does childhood experience of family victimization influence adulthood refusal of wife abuse? Evidence from rural Bangladesh
Source: PLoS One. 2021 Jun 3;16(6):e0252600. doi: 10.1371/journal.pone.0252600 (PMC8174681; doi:10.1371/journal.pone.0252600)
Supplement: S2 Table — (PDF) [file pone.0252600.s002.pdf]

S2 Table. Multivariate binary logistic regressions predicting different types of attitudinal refusal of wife abuse among women, *N*= 969

	Refused overall wife abuse			Refused emotional abuse			Refused physical abuse			Refused abuse on disobeying obligations			Refused abuse on challenging authority		
	OR	95% CI	P	OR	95% CI	P	OR	95% CI	P	OR	95% CI	P	OR	95% CI	P
**Childhood Abuses**															
**Emotional**															
None	4.00	1.35–11.89	0.012	1.36	0.58–3.15	0.478	1.15	0.54–2.43	0.724	1.43	0.66–3.11	0.364	3.10	0.98–9.01	0.038
Mild	2.76	1.57–11.89	<0.001	0.94	0.66–1.35	0.747	1.86	1.35–2.56	<0.001	1.56	1.12–2.19	0.010	2.46	1.27–4.17	0.001
Severe	1			1			1			1			1		
**Physical**															
None	0.98	0.54–1.77	0.939	0.98	0.60–1.60	0.938	1.01	0.68–1.51	0.954	0.93	0.62–1.41	0.745	0.98	0.55–1.75	0.951
Mild	1.26	0.78–2.04	0.354	1.49	1.02–2.15	0.037	1.12	0.82–1.54	0.477	1.42	1.02–1.99	0.040	1.27	0.80–2.03	0.310
Severe	1			1			1			1			1		
**Ethnicity**															
Garo	1.77	1.01–3.08	0.045	1.03	0.68–1.57	0.881	2.31	1.60–3.34	<0.001	0.70	0.47–1.05	0.083	1.85	1.08–3.19	0.026
Santal	2.27	1.34–3.83	0.002	0.92	0.63–1.36	0.687	1.71	1.22–2.39	0.002	0.48	0.33–.69	0.000	2.38	1.43–3.97	0.001
Bengali	1			1			1			1			1		
**Age in years**															
16-25	1.32	0.44–3.96	0.616	1.19	0.46–4.83	0.72	2.17	0.98–4.83	0.058	1.92	0.83–4.44	0.128	0.88	0.33–2.36	0.876
26-35	1.36	0.46–3.80	0.613	1.26	0.50–3.16	0.63	2.19	1.01–4.76	0.048	1.42	0.64–3.18	0.389	0.88	0.34–2.28	0.880
36-45	0.84	0.27–2.57	0.754	0.97	0.38–2.49	0.94	1.39	0.63–3.06	0.418	0.89	0.39–1.99	0.769	0.58	0.21–1.60	0.581
46-60	1			1			1			1			1		
**Schooling**															
Higher	3.19	1.38–7.36	0.01	1.06	0.60–1.89	0.833	2.28	1.33–3.92	0.003	1.86	1.04–3.31	0.04	3.10	1.39–6.90	0.006
Secondary	1.04	0.44–7.36	0.93	0.66	0.38–1.16	0.148	1.16	0.70–1.94	0.559	0.89	0.53–1.49	0.64	1.12	0.40–2.52	0.812
Primary	0.79	0.34–1.85	0.60	0.45	0.26–0.79	0.005	1.10	0.68–1.80	0.699	0.65	0.40–1.06	0.08	0.77	0.34–1.72	0.518
None	1			1			1			1			1		
**Monthly income**															
BDT 7000/above	1.17	0.62–2.20	0.64	0.91	0.53–1.56	0.732	1.07	0.65–1.73	0.801	1.04	0.62–1.74	0.894	1.22	0.66–2.25	1.216
Below BDT 7000	0.61	0.39–0.95	0.03	0.58	0.41–0.81	0.002	0.91	0.68–1.23	0.538	0.76	0.56–1.05	0.096	0.65	0.42–1.00	0.649
No income	1			1			1			1			1		
**Model summary**															
χ2 (16, N = 1929)	79.43***			45.18***			84.13***			86.82***			79.37***		
–Log Likelihood	688.287			1019.199			1258.196			1136.817			724.591		
