# Supplementary material for: Does childhood experience of family victimization influence adulthood refusal of wife abuse? Evidence from rural Bangladesh
Source: PLoS One. 2021 Jun 3;16(6):e0252600. doi: 10.1371/journal.pone.0252600 (PMC8174681; doi:10.1371/journal.pone.0252600)
Supplement: S3 Table — (PDF) [file pone.0252600.s003.pdf]

S3 Table. Multivariate binary logistic regressions predicting different types of attitudinal refusal of wife abuse among men, *N*= 960

	Refused overall wife abuse			Refused emotional abuse			Refused physical abuse			Refused abuse on disobeying obligations			Refused abuse on challenging authority		
	OR	95% CI	P	OR	95% CI	P	OR	95% CI	P	OR	95% CI	P	OR	95% CI	P
**Childhood Abuses**															
**Emotional**															
None	1.60	0.594–4.32	0.352	1.58	0.61–4.09	0.341	2.22	0.86–5.72	0.099	1.26	0.37–4.21	0.713	1.61	0.60–4.34	0.346
Mild	2.35	0.895–6.16	0.083	2.25	0.90–5.66	0.083	4.88	1.97–12.13	0.001	2.03	0.63–6.49	0.233	2.33	0.89–6.10	0.086
Severe	1			1			1			1			1		
**Physical**															
None	2.26	1.55–3.30	<0.001	2.06	1.42–2.98	<0.001	5.28	3.31–8.43	<0.001	4.61	2.66–7.98	<0.001	2.29	1.57–3.34	<0.001
Mild	1.27	0.87–1.86	0.215	1.16	0.80–1.69	0.425	3.05	1.97–4.73	<0.001	2.18	1.33–3.55	0.002	1.27	0.87–1.86	0.213
Severe	1			1			1			1			1		
**Ethnicity**															
Garo	2.22	1.57–3.12	<0.001	1.94	1.38–2.73	<0.000	1.55	0.97–2.47	0.067	0.99	0.58–1.70	0.967	2.22	1.57–3.12	<0.001
Santal	1.63	1.14–2.32	0.007	1.40	0.99–1.99	0.060	1.58	0.98–2.56	0.063	1.19	0.68–2.07	0.538	1.71	1.20–2.44	0.003
Bengali	1			1			1			1			1		
**Age in years**															
16-25	0.76	0.34–1.72	0.511	1.00	0.46–2.20	0.993	0.82	0.34–1.99	0.667	3.17	0.67–14.94	0.145	0.69	0.31–1.57	0.377
26-35	1.58	1.01–2.44	0.043	1.38	0.89–2.14	0.149	2.74	1.56–4.81	<0.001	1.30	0.68–2.51	0.430	1.48	0.95–2.29	0.082
36-45	1.28	0.85–1.91	0.234	1.17	0.78–1.74	0.451	2.10	1.27–3.46	0.004	1.18	0.65–2.14	0.593	1.21	0.81–1.81	0.357
46-60	1			1			1			1			1		
**Schooling**															
Higher	1.15	0.59–2.25	0.688	0.71	0.36–1.42	0.338	1.18	0.52–2.68	0.690	0.70	0.23–2.14	0.530	1.20	0.61–2.36	0.589
Secondary	0.83	0.44–1.55	0.551	0.46	0.24–0.88	0.019	1.60	0.74–3.47	0.233	0.60	0.21–1.71	0.336	0.87	0.46–1.63	0.657
Primary	0.99	0.55–1.81	0.983	0.60	0.32–1.12	0.109	1.47	0.71–3.02	0.299	0.60	0.22–1.64	0.323	1.10	0.60–2.01	0.754
None	1			1			1			1			1		
**Monthly income**															
BDT 7000/above	0.69	0.50–0.96	0.028	0.66	0.48–0.92	0.013	1.32	0.85–2.04	0.223	1.53	0.92–2.53	0.100	0.66	0.47–0.91	0.012
Below BDT 7000	1			1			1			1			1		
**Model summary**															
χ2 (16, N = 1929)	66.51***			56.52***			106.09***			43.62***			70.93***		
–Log Likelihood	1264.132			1270.32			784.261			635.785			1259.871		

Note for Monthly income: We merged ‘No income’ category with the ‘Bellow BDT 7000’ category as only two men did not earn an income during the survey.
