# Supplementary material for: Does childhood experience of family victimization influence adulthood refusal of wife abuse? Evidence from rural Bangladesh
Source: PLoS One. 2021 Jun 3;16(6):e0252600. doi: 10.1371/journal.pone.0252600 (PMC8174681; doi:10.1371/journal.pone.0252600)
Supplement: Attachment — Submitted filename: Response to Reviewers.docx [file pone.0252600.s005.docx]

**Responses to the comments:**

**Review Comments to the Author**

**Reviewer #1:**

**Comment-1:**

The authors have tackled an important public health problem that is crucial in primary prevention of intimate partner violence. However, I have several pertinent issues of concern with the study/paper, which I will outline below.

i) One of the main concern is on how the authors have defined the 2 main exposures (having multiple dichotomous variables that make it difficult to interpret their multivariate model results. In their description of the exposures they have defined the following categories “mild”, “moderate”, “severe” and “no victimisation”, which are assumed to be mutually exclusive categories. However, having multiple dichotomous variables (e.g moderate vs no victimization) as shown in Table 1 & 2 is not an efficient way of looking at the exposure. As Table 1 shows, participants classified as ‘mild’ include ‘moderate’ and ‘severe’ categories.
I would recommend that the authors define the exposures (emotional victimization & physical victimization) using 2 variables that have mutually exclusive categories (no victimization, mild, moderate and severe).

**Response-1:** Thank you for the very useful comments. We have re-categorized both the exposure variables into: ‘none,’ ‘mild,’ and ‘severe.’ Based on the similar pattern of occurrence, we merged mild/moderate emotional abuses and defined them as ‘mild.’ On the other hand, we merged moderate/severe physical abuses and defined them as ‘severe.’ Both the exposure variables (childhood emotional abuse and childhood physical abuse) are now well defined and easy to interpret. The changes are reflected in different paragraphs and tables, for example, pages 9-10, lines 215-235; page 14, line 319 (table 2); page 15, line 340 (table 3); and page 18, line 391 (table 4).

**Comment-2:**

ii) The authors should include a Table showing items used to measure the outcomes and also the main exposures. This would make it easier for the reader to understand how these have been measured and defined. As it is now, it is rather difficult to understand how the outcomes and exposures have been derived.

**Response-2:** Unfortunately, this will increase the length of the paper and the number of tables. We already have four tables. The items are discussed in defining the variables (see pages 8-10, lines 191-235). However, we included a table on the items used in the outcome and main exposure variables, respectively as supporting information file (see S1_Table). We also referred to them in the description of the study measures: page 8, line 195; and page 9, line 221.

**Comment-3:**

iii) The authors need to be consistent in how they refer to the main exposures. As it is, they talk of childhood victimization (which is a risk factor) and then switch to childhood non-victimization (as a protective factors). Most literature tend to look at childhood victimization as a being positively associated with acceptance of wife abuse, which is basically conversely same as looking at childhood non-victimization being positively associated with refusal of wife abuse (as defined by the authors). They need to be consistent in how they present these in the paper and Tables. Otherwise moving from one to the other is rather confusing. They may find that it is easier to look at victimization & acceptance attitude (going by the way they have presented their discussion and abstract).

**Response-3:** Thank you. We have tried to be consistent and revised the paper accordingly.

**Comment-4:**

iv) I understand that their paper is part of a bigger study which has both men and women. It would be interesting to see how their results differ (or not differ) if the authors performed subgroup analysis (by gender), especially the prevalence of ‘refusal of wife abuse.

**Response-4:** With this revision, we have now presented gender-disaggregated analysis/findings. For the refusal of wife abuse, see page 13, lines 297-302; page 13, line 304 (table 1). For childhood exposure to familial abuse, see page 13, lines 306-313; page 14, line 319 (table 2). Gender is also used as a controlled variable (moderator) in the final models: page 17, lines 387-390; page 18, line 391 (table 4). We also conducted subgroup analysis by gender and found that women are generally influenced by childhood emotional abuse, while men are mostly influenced by physical abuse: page 17, lines 387-390. We included these findings of multivariate analysis as supporting files: ‘S2_Table’ for women; ‘S3_Table’ for men.

**Comment-5:**

v) Other general comments that the authors need address are:
• They need to be a bit more succinct. They do not need to have a background & introduction. They need to cut down on the methods section especially their description of the Site of the study.

**Response-5:** We have now shortened the sections. We merged the introduction and the background and shortened it further. We also shortened the method section and description of study sites.

**Comment-6:**

• It is not clear if the sample size calculation presented in the paper is for this particular study or is for the main C-RCT. If it is for the main C-RCT, they need to make it clear. Otherwise it is difficult to link the parameters in their formula to the study objectives of this paper.

**Response-6**: Based on the findings of our pilot study, the sample size was estimated for the main C-RCT study, and this is now made more clear in the text. Please see page 8, lines 179-180.

**Reviewer #2:**

**Comment-7:**

Thank you for this paper which deals with a very critical topic, below I offer some feedback on the paper, I hope it is useful in strengthening the paper.

Abstract
I would suggest being clear from the beginning, and in the abstract who you’re referring to (men only or men and women) when you speak about “attitudinal refusal of wife abuse” I suggest saying ‘attitudinal refusal by women and men, of wife abuse”. Indeed, this needs to be clarified throughout, and I suggest later you may want to analyse and discuss by gender as well.

**Response-7:** We have now made it (i.e., who we are referring to - men only or men and women) clear in the abstract as well as throughout the paper. Now we clearly use ‘attitudinal refusal of wife abuse among men and women.’ In the paper, we analyzed the data by gender as well as tried to discuss the findings accordingly. We made the suggested changes in the abstract: page 2, lines 31-36. The results related to the refusal of wife abuse also now embraced with gender disaggregated findings: page 13, lines 297-302; page 13, line 304 (table 1); the exposure variable (childhood victimization) also included findings by gender: page 13, lines 306-313; page 14, line 319 (table 2).

Gender is also used as a controlled variable in the final models: page 17, lines 387-390; page 18, line 391 (table 4).

With this revision, we have also conducted subgroup analysis by gender and found that women are generally influenced by childhood emotional abuse, while men are mostly influenced by physical abuse: page 317, lines 387-390. We included these findings of multivariate analysis as supporting files: ‘S2_Table’ for women; ‘S3_Table’ for men.

**Comment-8:**

You indicate the % that “refused different types of wife abuse” – is this based on their views/attitudes, ie: do you think x is acceptable or not etc. Or is it based on whether the person had perpetrated any of these acts, ie: did you ask “have you ever done x” or “have you done x in the last 12 months” etc?? Please clarify for the reader. I am assuming as you refer to ARWA that this is ‘attitudes’ not actual actions. Please clarify this throughout the paper.

**Response-8:** Thank you for pointing this out. In this study, attitudinal refusal of wife abuse means a non-violent attitude toward wife abuse, not the actual action. We have now made this clear, both in the abstract and in the introduction: page 2, line 31; page 3, line 55.

**Comment-9:**

“We argue that the issues of childhood victimization should be brought to the center in the discourse” Please could you make this a much stronger recommendation, who would you call upon to do this and where? For example are you calling for more research, changes in policy???? And where? In Bangladesh or where???

**Response-9:** We have now made a more stronger recommendation in the abstract, page 2, lines 50-52, and in the discussion section: page 21, line 463-465; page 22, line 487-495.

**Comment-10:**

Introduction
As I stressed in my comments above please begin by indicating who you are speaking about “Attitudinal refusal of wife abuse (ARWA)…” – by whom? Men only or men and women.

**Response-10:** Please see our response no.8

We have now made it clear in the beginning of the paragraph (introduction). We use ‘attitudinal refusal of wife abuse among men and women’; please see page 3, line 55.

**Comment-11:**

Theory:
You make the following point “though little is known about why many adults who were exposed to childhood abusive events do not continue the same pattern with their partners” this is a very important point and I think it would be really good if you could add some reflections on what the literature is saying about this, even if there is not much. Then in the discussion you could see if your results and analysis are similar in anyway etc.

**Response-11:** We reviewed some literature on the issue (though we did not find many studies that give comprehensive explanation, see: page 5-6, lines 126-133; and also provided our speculation in the discussion in line with some of our findings, page 20- 21, lines 442-457.

Here, we have copied and pasted the whole paragraph included in the literature review section; page 5-6, as follows:

Studies have also consistently shown that childhood exposure to parental abuse is linked to their adulthood beliefs/behaviors related to wife abuse [23, 24], although little is known about why many adults who were exposed to childhood abusive events do not continue the same pattern with their partners [26]. It is possible that higher education and an awareness of the risks associated with abuse, a person’s self-esteem, relationship quality, meaningful victim support system, gender equitable environment, and good social control mechanism may de-motivate people to continue the abuse in adulthood even if they have experienced/internalized the abuse in their childhood [27-29]. A few studies based in Bangladesh have focused on the social learning of wife abuse as well as its social acceptance among community members [7, 30]. However, the association between childhood non-experience of various types of family victimization with attitudes in adulthood toward wife abuse is relatively overlooked in the previous studies conducted in Bangladesh. Hence, this study aims to enhance our understanding of what influence childhood learning and experiences of different abuses have on refusal of wife abuse in adulthood.

**Comment-12:**

Methods:
The methods section could be reduced quite a bit.

**Response-12:** We reduced the section accordingly.

**Comment-13:**

Study participants, you state clearly it was adults under 60, please indicate the youngest age for inclusion.

**Response-13:** We indicated it clearly; page 8, line 177. The same is reflected in the tables.

**Comment-14:**

“We excluded men/women with mental/physical disabilities.” Please indicate why they were excluded.

**Response-14:** We have now mentioned why we excluded them; see page 8, lines 177-179.

We added this sentence: Due to our lack of preparation and appropriate facilities, we excluded individuals with mental disorders and physical disabilities (e.g., deaf/mute).

**Comment-15:**

‘Social Acceptance of Wife Abuse Scale’ (SAWAS) – its great that you used a locally developed and validated scale.
The childhood exposure questions – please specify whether these covered both experience of and witnessing abuse (I assume it did?), and if it includes witnessing as well, perhaps include an example of one.

**Response-15:** We only assessed the actual experience of abuse during their childhood, not the witness of inter-parental abuse. We have now made it clear in the paper; page 9, lines 217-220.

**Comment-16:**

Results
I think it is important to discuss the results by men and women’s experiences – I think it is important to understand how these views and experiences differ. It would also be interesting to discuss the analysis by age, which would show, among other things, if there are any generational shifts occurring etc. Please can you undertake and present these results as well. Its interesting to see the effect of education and ethnicity and I imagine gender and age would also reveal interesting findings.

**Response-16:** We have now presented the gender disaggregated findings on both their views and childhood experiences; see: page 13, lines 297-302, 304 (table 1), 306-313; page 14, 319 (table 2). We have also presented the gender and age specific findings on the ARWA; page 14, lines 333-336. Multivariate findings also addressed the issues of gender and ethnicity: page 17, lines 381-390. Younger people refused physical abuse more than the older people, page 18, line 391(table 4).

**Comment-17:**

Discussion
I feel that discussions should not try to present all the data again, and should make a relatively smooth, yet powerful, read, so I think you might want to re-write the first paragraph, could you shorten it to make it more readable. The final sentence in the first paragraph does a great job of summarizing some results very effectively, perhaps you could consider something like that for the beginning of the paragraph

**Response-17:** We have re-written the first paragraph. Now, we only address the key findings in the beginning of the discussion; page 19, lines 395-401. We revised the paragraph accordingly.

**Comment-18:**

“These findings have offered a novel understanding of the issue and added to the current literatures that not all childhood abusive experiences equally contribute to the adulthood attitudinal refusal/acceptance of wife abuse” This is an important finding, and if we could also have this broken down by gender and age we would learn so much more!

**Response-18:** We have added a few lines on these findings as well as our understanding of this; page 20, lines 421-425. We observed that women’s attitudes were generally influenced by their childhood exposure to emotional abuse, while men’s attitudes were shaped by their experience of physical abuse; page 20, lines 423-425. We also discussed this according to formal educational attainment: page 20-21, lines 442-457.

**Comment-19:**

Please consider a final edit of the paper as there are still a few grammatical errors.

**Response-19:** The paper has been edited by a professional editor.

**Others**: The findings from multivariate are now presented more systematically: five models for the ARWA are presented separately one by one: page 16-17, lines 347-380.

**Editorial Comments to the Author**

Please include the following items when submitting your revised manuscript:

- A rebuttal letter that responds to each point raised by the academic editor and reviewer(s). You should upload this letter as a separate file labeled 'Response to Reviewers'.
- A marked-up copy of your manuscript that highlights changes made to the original version. You should upload this as a separate file labeled 'Revised Manuscript with Track Changes'.
- An unmarked version of your revised paper without tracked changes. You should upload this as a separate file labeled 'Manuscript'.

**Response:** Addressed

**Additional requirements**

1. Please ensure that your manuscript meets PLOS ONE's style requirements, including those for file naming. The PLOS ONE style templates can be found at

https://journals.plos.org/plosone/s/file?id=wjVg/PLOSOne_formatting_sample_main_body.pdf and

https://journals.plos.org/plosone/s/file?id=ba62/PLOSOne_formatting_sample_title_authors_affiliations.pdf

**Response:** Addressed

2. For studies involving humans categorized by race/ethnicity, age, disease/disabilities, religion, sex/gender, sexual orientation, or other socially constructed groupings, authors should: 1) Explicitly describe their methods of categorizing human populations, 2) Define categories in as much detail as the study protocol allows, 3) Justify their choices of definitions and categories, 4) Explain whether (and if so, how) they controlled for confounding variables such as socioeconomic status, nutrition, environmental exposures, or similar factors in their analysis.

**Response:** Addressed:

We elaborated the ethnic category, please see: page 10, lines 240-244. We also controlled for the possible confounding variables, please see: page 10, lines 237-239.

3. Please include captions for your Supporting Information files at the end of your manuscript, and update any in-text citations to match accordingly. Please see our Supporting Information guidelines for more information: http://journals.plos.org/plosone/s/supporting-information.

**Response:**  Addressed

4.Thank you for submitting the above manuscript to PLOS ONE. During our internal evaluation of the manuscript, we found some occurrences of overlapping text with the following previous publication(s), some of which you are an author, which needs to be addressed:

- https://journals.plos.org/plosone/article?id=10.1371/journal.pone.0236733

We would like to make you aware that copying extracts from previous publications, especially outside the methods section, word-for-word is unacceptable. In addition, the reproduction of text from published reports has implications for the copyright that may apply to the publications.

Please revise the manuscript to quote or rephrase the duplicated text and cite your sources for text outside the methods section. Please note that further consideration is dependent on the submission of a manuscript that addresses these concerns about the overlap in text with published work.

**Response:** Addressed throughout
