# Supplementary material for: Does childhood experience of family victimization influence adulthood refusal of wife abuse? Evidence from rural Bangladesh
Source: PLoS One. 2021 Jun 3;16(6):e0252600. doi: 10.1371/journal.pone.0252600 (PMC8174681; doi:10.1371/journal.pone.0252600)
Supplement: Attachment — Submitted filename: Response to Reviewers.docx [file pone.0252600.s006.docx]

**Thank you very much both the reviewers and the editor.**

**We have addressed all of your comments as follows:**

The changes are track-changed/highlighted as colored text throughout the paper.

**Comments**

**Journal Requirements:**

Please review your reference list to ensure that it is complete and correct. If you have cited papers that have been retracted, please include the rationale for doing so in the manuscript text, or remove these references and replace them with relevant current references. Any changes to the reference list should be mentioned in the rebuttal letter that accompanies your revised manuscript. If you need to cite a retracted article, indicate the article’s retracted status in the References list and also include a citation and full reference for the retraction notice.

**Response:**

We have removed the following reference and replaced it with an original paper:

“25. Gelles RJ. Spouse Abuse - Theoretical Explanations. Marriage and Family Encyclopedia: Net Industries; 1995 [October 15, 2007]. Available from: <http://family.jrank.org/pages/1629/Spouse-Abuse-THEORETICAL-EXPLANATIONS.html>.

# Replaced with: “25. Gelles RJ. Family Violence. Annu Rev Sociol. 1985; 11: 329-346.”

**Comments**

**Reviewer #1:**

The authors have made some substantial revision and incorporated most of the recommendation from the previous comments and suggestions. I have a few more comments as follows:
1) Authors have indicated that the sample size formula indicated in the paper is for the main study which they have described as a cluster-randomised control trial. This makes their explanation even more confusing. The sample size calculation formula quoted in the paper does not seem to be for a cluster-randomised control trial that is supposed to assess effect of an intervention, but rather for a prevalence study. Could the authors clarify this.

**Response 1:**

We have further revised the sentences and made it clear (see page 8, line: 213-217) as:

“By taking into account the findings from our pilot study conducted in selected villages, we computed the sample size for the baseline survey. Considering the prevalence of sexual abuse in a village (*p*=0.167), the minimum sample size was estimated at 1,854 using a formula (n = z^2^_α/2_ p(1‑p)/E^2^…”

**Our further replies:**

Regarding the effects of community mobilization intervention on domestic violence, we lack previous data in the context of Bangladesh. Therefore, in order to estimate the required minimum sample size, we used the current prevalence of DV in a study village. We tried to maximize the required sample size and that is why we used the lowest prevalence of sexual abuse.

Now we have data on our intervention effects in a few Bengali villages. It shows that the prevalence of emotional DV in our intervention sites is 72% while it is 81% in the control areas. Thus if we estimate our minimum sample size for each group, it should be 472 for 95% confidence interval and 90% given power (e.g. using the formula: sample size: n= (Z_1-β_+Z_1-α/2_)^2^[(R+1)]−p_2_(R^2^+1)/p_2_(1-R)^2^ ; where n=sample size in each group, p_1_=proportion in intervention group, p_2_=proportion in control group, R= relative risk, Z_1-β_ = Z score for 90 percentile, and Z_1-α/2_ = Type 1 error set at α=0.05. Adjusting with 95% response rate in the intervention, thus it should be enough to have total 500+500= 1000 samples from the villages. Thus, we believe that our 1,929 samples should be representative for both prevalence and intervention studies.

**Comments**

2) Tables 1 and 2 has showed significant differences in both the outcomes and main exposures between men and women. Adjusted for sex of participant in the model may mask the relationship between childhood victimization and the outcome. It is also common practice in violence analysis to look at men and women separately. Authors need to discuss the differences in outcome and main exposure between men and women in the context of Bangladesh as well as in the context of existing knowledge.

**Response 2:**

We are keeping the current model as it helps us to understand the gender differences in the attitudinal refusal of wife abuse, though we are aware of the limitations of this strategy. However, we have also presented the findings for men and women separately in S2-3 Tables.

Considering the context of Bangladesh as well as existing knowledge, we also discussed the issue why only few women attitudinally refused wife abuse compared to men as follows:

“The study further shows that men are more likely to refuse all types of wife abuse compared to women in rural Bangladesh. These results are in line with the findings of previous studies showing that women uphold more conservative gender ideology than their male counterparts in low-income patriarchal societies like Bangladesh [11, 12, 45]. This is possible because both men and women can contribute to accept, maintain and reinforce the patriarchal social order. As a ‘subordinated gender’, women’s acceptance/reinforcement of these patriarchal traditions makes sure the continuation of wife abuse/male domination over women [12, 54]. This indicates that most of the women are not so aware of their rights.”

(See page 25, Line: 496-503)

**Comments**

3) Authors need to clarify further how the derived the 2 outcomes (refusal of emotional abuse and refusal of physical abuse) from the 6 items. It is clearer now how they derived the other 3 outcomes but it is not clear how participants’ responses to the 6 items were used to derive the 2 outcomes. Perhaps they could include a footnote as they have done for the exposure definition table.

**Response 3:**

We have further clarified this in the method section as follows (see page 9, line: 237-241):

“In order to compute the variables (refusal of emotional abuse and refusal of physical abuse), we recoded the responses. We derived the refusal of emotional abuse variable after merging ‘yes – emotional abuse’ and ‘yes – both emotional and physical abuse’ responses. Then again, we got the refusal of physical abuse by adding ‘yes – physical abuse’ and ‘yes – both emotional and physical abuses’ responses.”

We have also added a footnote under Table 1 (see page 14, line: 348-352).

**Comments**

4) Considering that the focus of the paper is on the relationship between childhood victimization and attitudes towards abuse of wife, the authors should present the final table of results that highlight this objective. I recommend that they present the unadjusted and then the adjusted ORs for the 2 exposures. They could then assess whether it is necessary to control for all the variable they have included in the adjusted model, considering that the number of participants in “severe abuse” categorical for some of the outcomes is small.

**Response 4:**

We have now presented unadjusted ORs in the Table 3 (see page 22).

In the adjusted models, we have only included the significant variables (see Table 4) (page 23).

**Comments**

5) Table 3 should also be stratified by sex to complement Tables S2 and S3.

**Response 5:**

Part of Table 3, Table 1 and Table 2 are now merged and presented as Table 1 (page 14).

The Table 1 is now presenting sample socio-demographic characteristics, and the prevalence of outcome variables as well as the prevalence of exposure variables among the samples.

Table 3 is now presented as Table 2 (page 17-18).

All of these tables (Table 1 and Table 2) are now stratified by sex.

**Comments**

6) Have the authors looked at the intersection of childhood emotional abuse and physical abuse, considering that these are not mutually exclusive experiences.

**Response 6:**

We have found no significant interactions between these two variables. Thank you so much.

**Comments**

7) Could the authors check why they are getting weird CIs for income in S3. A table of frequencies (as suggested in point 5) would assisting in understanding what is happening here.

**Response 7:**

We have corrected this. It was related to a category of income, where we had only two samples. They could be the outliers in the male samples; e.g., two men did not have any income during our fieldwork. So, we have merged ‘No income’ and ‘Bellow BDT 7000” categories (see S3 Table).

**Comments**

**Reviewer #2:**

Thank you for revising the paper in line with the peer review you received. I think the paper is greatly improved and presents a very important argument, the discussion is very well structured and makes many important points. However, I believe there are still a few small areas in which the paper could be improved, I hope my comments are helpful.

Introduction:
The first sentence reads as “Attitudinal (not actual act) refusal of wife abuse (ARWA) among men and women is a crucial factor that practitioners attempt to enhance in the society for the primary prevention of intimate-partner violence (IPV) in many low- and lower-middle income countries” I think you can delete the brackets (Not actual act) and make this point clearly in the third sentence, as the first sentence is written currently with the brackets is a little tricky to follow. Perhaps you could add to the end of your third sentence “… and focuses on attitudes and not actual acts of abuse.”

**Response 8:**

We made these changes (see page 3, line 59-62).

The third sentence now read as “As the attitudinal refusal of wife abuse focuses on a mindset and not actual acts of abuse, it is a crucial aspect of IPV. Refusal/acceptance of wife abuse is also a widely talked about issue in the discourse of the primary prevention of IPV against women.”

**Comments**

“In contrast, the refusal of wife abuse signifies the notions where both the male and the female members of a society reject the abuse [5]. This refers to the attitudinal stand where people reject wife abuse. ARWA is crucial in preventing IPV.” I think these sentences could be deleted, they are obvious and feel like you are over-stating the case and becoming repetitive.

**Response 9:**

We deleted these sentences as the theme appears in the previous sentences (page 3, line 69).

**Comments**

” Violent husbands often try to defend the abuse by pointing out their ‘wives’ faults’” I’m not sure they are ‘defending their abuse’ as they probably don’t see it as abuse – I think you should rather talk about ‘explaining their actions’ – this does not imply that they think they need to defend anything nor that they think of it as abuse.

**Response 10:**

We revised the sentence (see page 4, line 107) as:

“Violent husbands often try to explain their actions by pointing out their ‘wives’ faults’”

**Comments**

Theoretical perspective:
The first paragraph is repetitive, please remove the repetition. Perhaps it would be useful to go through the paragraph and see how many ideas/points you have in the paragraph and where you repeat the same idea or point, and then see what can be removed.

**Response 11:**

We have now removed all the repetitions from the paragraph (see page 5: line 120-134).

**Comments**

“however, a recent study indicates that there may be at least four dimensions where a person may refuse wife abuse on the grounds of: the wife failing to fulfill her family obligations, for challenging male family authority, physical abuse, and emotional abuse [31].” I think this sentence is wrong – I believe you are listing the reasons people use to justify men abusing their wives, I don’t think it is used as grounds to refuse abuse. I think you need to re-write this something like… “however, a recent study indicates that there may be at least four dimensions where a person may justify wife abuse on the grounds of: the wife failing to fulfill her family obligations, for challenging male family authority, physical abuse, and emotional abuse.”

**Response 12:**

We have revised the sentences (see page 6, line 163-167) as:

“however, a recent study indicates that there may be at least four dimensions where a person may justify/refuse wife abuse [31]. The four dimensions are as follows: acceptance/refusal of emotional abuse, acceptance/refusal of physical abuse, acceptance/refusal of abuse on the grounds of wife’s disobeying family obligations, and acceptance/refusal of abuse on wife’s challenging male family authority, [31].”

**Comments**

Control variables:
You note that “They [Bengali’s] are traditionally patriarchal. In contrast, the Garos are an ethnic minority community upholding matrilineal traditions, whereas the Santals are a patriarchal indigenous community living in Bangladesh”. I believe this is a very important point about the communities you are studying, and it is important to understand whether differences occur for the matrilineal communities, therefore I think you should also mention this when describing the study sites earlier.

**Response 13:**

We added this under the section ‘Sites under study’ (see page 7, line: 200-207):

The text can be read now as follows:

“The mainstream *Bengali* and the ethnic minority *Santal* villages are patriarchal whereas the ethnic *Garo* society follows matrilineal (matriarchal) traditions. In the *Garo* villages, women are traditionally treated as the heads of the households; and all of the family properties are also passed down to the female line. On the other hand, men own most of the household properties as well as they rule the families in the both *Bengali* and *Santal* villages. Due to their veiled seclusion, women’s physical mobility is quite low in the *Bengali* villages. However, women enjoy greater freedom to move in public spheres in the both *Garo* and *Santal* villages.”

**Comments**

Results:
I would suggest you consider moving the socio-demographic details of the participants to the beginning of the results, It is often good to begin by describing your participants (with the socio-demographic detail) and then move into other results.

**Response 14:**

We have now started by presenting the socio-demographic details (page 13, line 336).

Tables are also now rearranged accordingly. Please see: page 14, 17, 22 and 23)

**Comments**

“Our findings also reveal that respondents from ethnic communities are more likely to refuse most of the wife abuses than the mainstream communality”, please clarify who you are referring to when you say ‘ethnic communities’, I assume you mean the two communities who are no Bengali? Please clarify.

**Response 15:**

We have revised the sentence (page 26, line 504-506) as:

“Our findings also reveal that respondents from the *Garo* and *Santal* ethnic minority communities are more likely to refuse most types of wife abuse than the mainstream *Bengali* communality.”

**Comments**

Discussion:
“Specifically, our study indicates that the childhood exposure to mild emotional abuse might increase the attitudinal refusal of wife abuse in adulthood” this sentence is a bit confusing – am I right to think that exposure to ‘mild emotional abuse’ leads to adults being more likely to accept wife abuse, ie not to refuse wife abuse? Please clarify the sentence.

**Response 16:**

We have clarified the sentence (see page 25, line 485-487) as:

“Regarding the childhood victimization of emotional abuse, our study indicates that those who experienced mild emotional abuse during childhood are more likely to refuse the wife abuse in adulthood than those who experienced severe emotional abuse.”

We also surprised with this finding, however we provided an explanation (page 25, line 347-495)

**Comments**

Grammar:
I note you had the paper reviewed for grammar, and I think it is reading much better, but I think it could benedict from one more edit, there are places where the sentence structure is difficult to follow and this can confuse the reader, for example in the results you say “The attitudinal refusal of different wife abuses also appeared to be less among women than men” I believe it might read better if written as “The attitudinal refusal of different FORMS OF wife abuse appeared to be LOWER among women than men” Another example is that in the results the authors shift to using the acronym ARWA a lot, I would suggest rather writing ‘attitudinal refusal of wife abuse’ I think using the acronym interrupts the flow of the sentences, however, I see that this may become very repetitive, so perhaps the sentences could be restructured to address repetition if needed.

**Response 17:**

We have gone through the paper again for further language edit and we tried to address the issues you raised. We also replaced the acronym ARWA with its full form. Thank you.
