# Supplementary material for: Does childhood experience of family victimization influence adulthood refusal of wife abuse? Evidence from rural Bangladesh
Source: PLoS One. 2021 Jun 3;16(6):e0252600. doi: 10.1371/journal.pone.0252600 (PMC8174681; doi:10.1371/journal.pone.0252600)
Supplement: Attachment — Submitted filename: Response to Reviewers.docx [file pone.0252600.s007.docx]

**Thank you very much.**

**We have addressed all of the comments as follows:**

The changes are track-changed/highlighted as colored text throughout the paper.

6. Review Comments to the Author
**Reviewer #1:**

The authors have made some substantial revision and incorporated most of the recommendation from the previous comments and suggestions. However, I still have a few more comments, suggestions and request for clarification as follows:

**Comment i)**

i) With the sample consisting of both men and women and also from 2 different traditions (matrilineal and patrilineal), I wonder how the 2 (sex of participant and type of tradition) are interacting. Did the authors look at the interaction between sex of participant and type of tradition or ethnic grp?

**Response i)**

We tested these interaction effects, and found that none of them were statistically significant.

| **Tests of Model Effects** | | | |
| --- | --- | --- | --- |
| Source | Type III | | |
|  | Wald Chi-Square | df | Sig. |
| (Intercept) | 221.014 | 1 | .000 |
| Sex | 242.694 | 1 | .000 |
| Tradition | 12.037 | 1 | .001 |
| Tradition * Sex | .008 | 1 | .927 |
| Dependent Variable: Refusal of any wife-abuse  Model: (Intercept), Sex, Tradition, Tradition * Sex | | | |

| **Tests of Model Effects** | | | |
| --- | --- | --- | --- |
| Source | Type III | | |
|  | Wald Chi-Square | df | Sig. |
| (Intercept) | 83.916 | 1 | .000 |
| Sex | 162.891 | 1 | .000 |
| Tradition | 2.932 | 1 | .087 |
| Tradition * Sex | 2.738 | 1 | .098 |
| Dependent Variable: Refusal of emotional abuse  Model: (Intercept), Sex, Tradition, Tradition * Sex | | | |

| **Tests of Model Effects** | | | |
| --- | --- | --- | --- |
| Source | Type III | | |
|  | Wald Chi-Square | df | Sig. |
| (Intercept) | 231.255 | 1 | .000 |
| Sex | 152.465 | 1 | .000 |
| Tradition | 15.713 | 1 | .000 |
| Tradition * Sex | 1.366 | 1 | .243 |
| Dependent Variable: Refusal of physical abuse  Model: (Intercept), Sex, Tradition, Tradition * Sex | | | |

| **Tests of Model Effects** | | | |
| --- | --- | --- | --- |
| Source | Type III | | |
|  | Wald Chi-Square | df | Sig. |
| (Intercept) | 452.735 | 1 | .000 |
| Sex | 103.954 | 1 | .000 |
| Tradition | .520 | 1 | .471 |
| Tradition * Sex | .159 | 1 | .690 |
| Dependent Variable: Refusal on disobeying obligations  Model: (Intercept), Sex, Tradition, Tradition * Sex | | | |

| **Tests of Model Effects** | | | |
| --- | --- | --- | --- |
| Source | Type III | | |
|  | Wald Chi-Square | df | Sig. |
| (Intercept) | 198.138 | 1 | .000 |
| Sex | 238.883 | 1 | .000 |
| Tradition | 12.885 | 1 | .000 |
| Tradition * Sex | .027 | 1 | .868 |
| Dependent Variable: Refusal on challenging male-authority  Model: (Intercept), Sex, Tradition, Tradition * Sex | | | |

**Comment ii)**

ii) Could the authors clarify in the paper how a participant was selected in a household where both a man and woman were available?

**Response ii)**

Thank you for asking this. We further clarified and added to the manuscript as follows:

“At first, we identified the study villages. After selecting a village, we collected an updated list of households. Each of the villages consists of roughly 100−300 households. We randomly identified the first household in a selected village. In order to form a cluster, we then included 81 additional households close to the first household. These households were the most physically nearest households from the household we selected at first. To do so, we conducted a quick household mapping in the area. After that, we randomly assigned half of the households for male respondents and the other half for female respondents. As a final point, we approached one respondent from a household for face-to-face interviews. If a household had more than one eligible respondent, we randomly selected one.”

**Line: 191-199**

**Comment iii)**

iii) I would like the authors to clarify more explicitly either in the text or in Table S1 how they are classifying the 4 responses for each item into refusal and non-refusal for the two outcomes (refusal of emotional abuse and refusal of physical abuse). I still find it hard to understand how these 2 outcomes were derived. For example, when they are looking at refusal of emotional abuse, does the ‘refusal group’ include the ‘yes, physical abuse’ ( does it include those that responded 0=no abuse to all the 6 items plus those that responded 2=yes, physical abuse to all the 6 items?

**Response iii)**

We have further clarified this in Table S1.

The table represents how we have recoded the data and classified the variables.

Some of the refusal of emotional abuse group may not refuse physical abuse

Please see Table S1 where we clarified how we have derived these variables.

**Comment iv)**
iv)In line 287-289, the authors have defined the OR with respect to a continuous exposure when none of the exposures used in their models are continuous. I would recommend that they just state that ORs were used to report associations between exposures and outcomes.

**Response iv)**

Thank you so much for letting us noticed this. We have revised the sentence as follows:

“Coefficients of logistic regression with log link function, exp*(β),* produced Odds Ratio (OR), which facilitated the explanation of regression results. We used ORs to report associations between exposures and outcomes.”

**Line: 292-293**

**Comment v)**

v) In line 190, instead of “To avoid for possible non-consent and drop out…” they should write “To account for …..”

**Response v)**

We have addressed this as follows:

To account for possible non-consent and drop out, we created a sample pool, including 10% over samples.

**Line: 188-189**

**Comment vi)**

vi) In line 348, change “not-victims’ to ‘non-victims’

**Response vi)**

We have corrected this. Thank you.

**Line: 352**

**Comment vii)**

vii)Could the authors discuss the any differences in associations between outcomes and ethnicity between men and women ( Table S2 & S3).

**Response vii)**

We have discussed these gender differences in associations between outcomes and ethnicity in the three sections of the manuscript as follows:

**Result section: line 420-432**

“Though the refusal of wife abuse was usually higher among the ethnic minority *Garo* and *Santal* communities than the mainstream *Bengali* community, we also observed gender differences in associations between outcomes and ethnicity (see S2 and S3 Tables). The refusal of overall wife abuse, refusal of emotional wife abuse, and refusal of wife abuse on challenging male authority appeared to be higher among Garo and Santal men than the Bengali men. However, there were no significant differences in the attitudinal refusal of physical wife abuse and the refusal of wife abuse on disobeying family obligations between the male respondents of different communities. On the other hand, women from both Garo and Santal communities attitudinally refused more overall wife abuse, physical abuse, and abuse on challenging male authority than the women from the Bengali community. At the same time, there were no significant differences in the refusal of emotional abuse between the women from different ethnicities. Conversely, the refusal of wife abuse on disobeying family obligations appeared to be lower among Garo and Santal women than the Bengali women.”

**Discussion section: line 498-594**

Our findings also reveal that respondents from the *Garo* and *Santal* ethnic minority communities are more likely to refuse most types of wife abuse than the mainstream *Bengali* communality. Although a previous study [12] indicated similar findings, our current study further shows that these ethnic minority communities did not refuse wife abuse on the ground of the wife's disobeying family obligations more than the mainstream *Bengali* community. It also reveals that there are gender differences in the associations between outcomes and ethnicity. The feature of the refusal of physical wife abuse and the refusal of wife abuse on disobeying family obligations appears similar among the male respondents of different communities. On the other hand, women of different ethnicities do not differ from each other in refusing emotional wife abuse. Conversely, the refusal of wife abuse on disobeying family obligations appears lower among Garo and Santal women than the Bengali women.

**Limitation section: line 535-539**

It was expected that the attitudinal refusal of wife abuse would be higher among the matrilineal communities than the patrilineal communities. However, we had a surprising finding, and we lack a proper explanation of why women from ethnic communities refused less abuse on the ground of the wife's disobeying family obligations than mainstream Bengali women. An explorative qualitative study may answer these research questions thoroughly.
